# A huge intraductal papillary mucinous carcinoma of the bile duct treated by right trisectionectomy with caudate lobectomy

**DOI:** 10.1186/1477-7819-7-93

**Published:** 2009-12-05

**Authors:** Won-Joon Sohn, Sungho Jo

**Affiliations:** 1 Department of Surgery, Dankook University College of Medicine, San#29, Anseo-dong, Dongnam-gu, Cheonan-si, Chungnam, 330-714, Korea

## Abstract

**Background:**

Because intraductal papillary mucinous neoplasm of the bile duct (IPMN-B) is believed to show a better clinical course than non-papillary biliary neoplasms, it is important to make a precise diagnosis and to perform complete surgical resection.

**Case presentation:**

We herein report a case of malignant IPMN-B treated by right trisectionectomy with caudate lobectomy and extrahepatic bile duct resection. Radiologic images showed marked dilatation of the left medial sectional bile duct (B4) resulting in a bulky cystic mass with multiple internal papillary projections. Duodenal endoscopic examination demonstrated very patulous ampullary orifice with mucin expulsion and endoscopic retrograde cholangiogram confirmed marked cystic dilatation of B4 with luminal filling defects. These findings suggested IPMN-B with malignancy potential. The functional volume of the left lateral section was estimated to be 45%. A planned extensive surgery was successfully performed. The remnant bile ducts were also dilated but had no macroscopic intraluminal tumorous lesion. The histopathological examination yielded the diagnosis of mucin-producing oncocytic intraductal papillary carcinoma of the bile duct with poorly differentiated carcinomas showing neuroendocrine differentiation. The tumor was 14.0 × 13.0 cm-sized and revealed no stromal invasiveness. Resection margins of the proximal bile duct and hepatic parenchyma were free of tumor cell. The patient showed no postoperative complication and was discharged on 10^th ^postoperative date. He has been regularly followed at outpatient department with no evidence of recurrence.

**Conclusion:**

Considering a favorable prognosis of IPMN-B compared to non-papillary biliary neoplasms, this tumor can be a good indication for aggressive surgical resection regardless of its tumor size.

## Background

Biliary intraductal neoplasms occur in both intrahepatic and extrahepatic bile ducts and are proposed to have two types; a flat and a papillary type [1,2]. Neoplastic lesions contained in a flat type are biliary intraepithelial neoplasia (BilIN) and non-papillary cholangiocarcinoma. A papillary type includes intraductal papillary mucinous neoplasm of the bile duct (IPMN-B) with malignancy potential, or biliary papilloma(tosis) and papillary cholangiocarcinoma. Contrary to well-documented flat type neoplasms, IPMN-B is relatively rare and a recently emerged disease entity. Although not a few reports on IPMN-B have been accumulated since the first description of mucus producing papillary cholangiocarcinoma by Isogai et al., in 1986 [3], there are still controversies on several aspects of IPMN-B and its concept is in process of establishment. Papillary cholangiocarcinoma is believed to show a better clinical course than non-papillary cholangiocarcinoma [4-9], as malignant intraductal papillary mucinous neoplasm of the pancreas (IPMN-P) has a better prognosis than pancreatic ductal adenocarcinoma. Therefore, it is important to make a precise diagnosis of IPMN-B and to perform complete surgical resection. We herein report a case of an extremely huge intraductal papillary mucinous cholangiocarcinoma successfully treated by right trisectionectomy with caudate lobectomy and extrahepatic bile duct resection.

## Case presentation

A 43-year-old male patient was referred for a huge cystic tumor of the liver, which was detected in abdominal ultrasonography (US) performed for right upper quadrant discomfort in local clinic. He was admitted to our hospital for further evaluation. He had no other symptom or past medical history of liver disease. He did not smoke and was a social drinker. His physical examination on admission revealed vaguely enlarged liver palpable three finger breadths below the right costal margin without definite tenderness; otherwise, there was no significant finding including vital sign.

**Figure 1:**
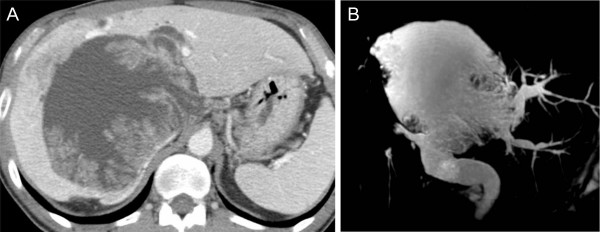
**Axial image of enhanced computed tomography shows a bulky cystic mass with multiple internal papillary projections involving the right hemiliver and left medial section (A)**. Magnetic resonance cholangiography (MRC) reveals a marked aneurysmal dilatation of the bile duct itself of the left medial section and a diffuse dilatation of the extrahepatic bile duct (B).

**Figure 2:**
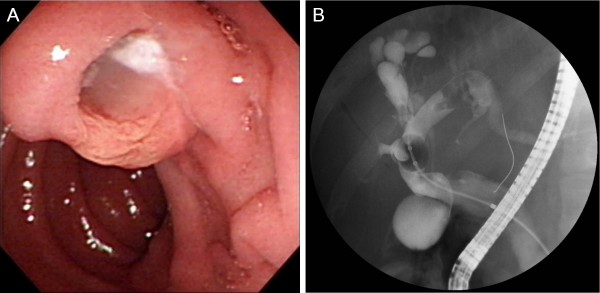
**Duodenal endoscopy demonstrates mucin expulsion from the patulous ampullary orifice (A)**. The findings of endoscopic retrograde cholangiogram (ERC) are similar to those of MRC, but ERC additively shows amorphous intraluminal filling defects corresponding to mucin and papillary tumors within the dilated bile ducts (B).

Initial laboratory values included WBC of 9,490/μL, albumin of 4.2 g/dL, total bilirubin of 0.8 mg/dL, AST/ALT of 58/65 IU/L, alkaline phosphatase of 387 IU/L, γ-GTP of 764 IU/L, α-FP of 0.7 ng/mL, CEA of 3.1 ng/mL, CA 19-9 of 21.1 U/mL. Liver computed tomography (CT) and magnetic resonance imaging showed marked dilatation of the left medial sectional bile duct (B4) resulting in a bulky cystic mass with multiple internal papillary projections. This cystic mass was so huge as to displace the parenchyma of most right hemiliver, left medial section, and caudate lobe, compressing both portal pedicles, main hepatic veins, and inferior vena cava (Figure 1A, 1B). No intrahepatic and extrahepatic metastasis was found. Duodenal endoscopic examination demonstrated a patulous ampullary orifice with mucin expulsion and endoscopic retrograde cholangiogram confirmed a marked aneurysmal dilatation of B4 with luminal filling defects (Figure 2A, 2B). These findings suggested IPMN-B with malignancy potential and prompted us to plan a curative extended major hepatectomy and extrahepatic bile duct resection. Liver volumetry was undertaken and the functional volume of the left lateral section was estimated to be 45%.

**Figure 3:**
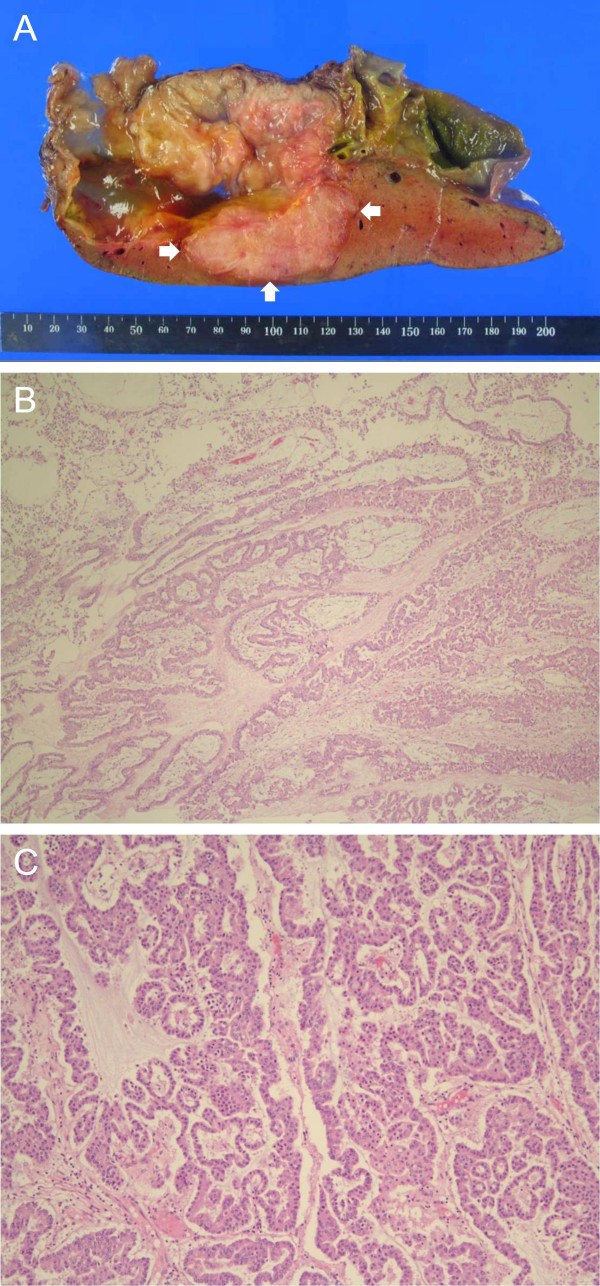
**The macroscopic appearance of the transected specimen (A) reveals a cystic dilatation of the intrahepatic bile ducts with intraluminal mucin and multiple papillary tumors**. Arrows indicate a poorly differentiated carcinoma with neuroendocrine differentiation abutting on the main tumor. Histopathological examination (B) demonstrates papillary structures without stromal invasion (hematoxylin and eosin ×40). This oncocytic type papillary cholangiocarcinoma (C) shows a columnar lining with abundant oxyphilic granular cytoplasm with intraepithelial lumina, which gives rise to a cribriform pattern of growth (hematoxylin and eosin ×200).

Neither ascites nor peritoneal metastatic nodule was detected during initial intraperitoneal exploration. A single enlarged regional lymph node was encountered and excised for frozen section, and the result was free of tumor cell. Therefore, further dissection of lymph node was not performed. The tumor was adherent to but detachable from the left portal pedicle, the inferior vena cava, and the left hepatic vein. The remnant left lateral sectional bile ducts were also dilated but had no macroscopic intraluminal tumorous lesion, which was ascertained by intraoperative cholangioscopy. Consequently the planned right trisectionectomy with caudate lobectomy and extrahepatic bile duct resection could be successfully performed. Macroscopic examination of the resected specimen revealed a cystic dilatation of the intrahepatic bile ducts with intraluminal mucin and multiple papillary tumors (Figure 3A).

Postoperative PT/INR level was normal and the values of total bilirubin and AST/ALT were peak on postoperative date (POD) 1, 6.7 mg/dL and 149/71 IU/L, respectively; after that they were gradually normalized. The histopathological examination for the resected specimen yielded the final diagnosis of mucin-producing oncocytic intraductal papillary carcinoma of the bile duct and revealed no stromal invasiveness (Figure 3B, 3C). The tumor was 14.0 × 13.0 cm-sized and involved the distal portion of the left intrahepatic duct and B4. No additional intraductal tumor was found in the right intrahepatic and extrahepatic bile duct. Resection margins of the proximal bile duct and hepatic parenchyma were free of tumor cell. The tumor showed no adenoma component and ovarian like stroma. There were another two (6.0 and 2.3 cm) poorly differentiated carcinomas showing neuroendocrine differentiation and microvascular invasion. The larger carcinoma (Figure 3A) was attached to the main tumor on the anterior side (segment 8), and the smaller one was 0.2 cm apart from the main tumor and 1.5 cm posterolateral to the larger one. They were immunohistochemically reactive for neuron-specific enolase, chromogranin, and synaptophysin. Dissected regional lymph node was confirmed to be without tumor cell. The patient showed no postoperative complication and was discharged on POD 10. He has been regularly followed at outpatient department with no evidence of recurrence.

## Discussion

Two types of biliary intraductal neoplasms preceding invasive cholangiocarcinoma have been identified so far; a flat type neoplastic lesion called BilIN, which develops into non-papillary cholangiocarcinoma, and a papillary type called IPMN-B with malignancy potential [1,2]. IPMN-B comprises a histological spectrum that ranges from benign to malignant: adenoma, borderline tumor, carcinoma in situ, and invasive carcinoma [9-11]. The current WHO classification and some authors recognize biliary papillomatosis, as well as BilIN, or biliary epithelial dysplasia, as precursor lesion of cholangiocarcinoma [12-16]. Biliary papillomatosis is a rare disease characterized by multiple microscopic papillary adenomas, therefore it could be regarded as benign or borderline form of IPMN-B [13]. Although the tumor of our case was extremely large and most papillary projections were carcinoma without adenoma component, invasion was confined to the epithelium (carcinoma in situ). Once papillary cholangiocarcinoma shows stromal invasiveness, its prognosis is as similarly poor as non-papillary cholangiocarcinoma. On the other hand, our case could be classified as an intraductal growth type intrahepatic cholangiocarcinoma (ICC). This type, among three gross types of ICC, is an entity described in recent years and designated as mucin-producing ICC or intrahepatic IPMN-B [4,8,11,17,18], which corresponds to a malignant form of IPMN-B, or papillary cholangiocarcinoma; the other mass-forming and periductal infiltrative types, which are more common and typical, could be called non-papillary cholangiocarcinoma.

Accumulated radiologic and endoscopic information on IPMN-B makes a diagnosis not so difficult. Specific findings of CT or US and cholangiograms, such as marked biliary dilatation and amorphous filling defects within the dilated bile ducts, could raise suspicion and simultaneous endoscopic demonstration of mucobilia is specific for the diagnosis of IPMN-B [7,8,18]. Before operation, we could highly suspect IPMN-B and plan an aggressive surgery. Yeh et al., classified cholangiographic types stratified by histologic grading in patients with IPMN-B and proposed tailoring the optimal management strategy based on the cholangiographic spectrum [10]. According to this study, our case was preoperatively suspected as IPMN-B type 3 or 4 (in situ or invasive adenocarcinoma) and cholangiographic type IIA or IIB (intrahepatic polypoid or cystic neoplasia with or without involvement of extrahepatic bile duct); in this situation, an aggressive resection should be aimed.

Because IPMN-B is considered to have a favorable prognosis after complete surgical resection, an aggressive surgery deserves to be recommended regardless of tumor size and extent [4-10]. The extremely huge tumor of the present case could be safely resected through right trisectionectomy with caudate lobectomy, not through other major hepatectomy; although the tumor was located in the central portion of the liver, it was tightly compressing the hepatic inflow and outflow vessels and inferior vena cava as well as displacing considerable portion of the right hemiliver, left medial section, and caudate lobe; furthermore, the functional volume of the future liver remnant, the left lateral section, was estimated to be 45%. Additionally, extrahepatic bile duct resection was inevitably required due to involvement of the tumor in the hilar bifurcation of the bile duct. Resultantly we could successfully get tumor-free margins without postoperative complications.

The unique pathological feature of our case was the two poorly differentiated carcinomas showing neuroendocrine differentiation. These tumors were not considered as metastatic lesion from an extrahepatic origin but instead were recognized as direct protrusion from the main tumor or connected daughter nodules; because they were abutting on the main cystic tumor and both the main tumor and the two adjacent carcinomas showed positivity in specific staining for neuroendocrine differentiation. The clinical significance of the two poorly differentiated carcinomas has not been determined but may be related to recurrence; they showed microvascular invasion. There has been no report on IPMN-B with accompanying carcinoma of neuroendocrine differentiation or intrahepatic daughter nodules.

IPMN-B is subdivided on the basis of histology and mucin gene protein (MUC1, MUC2, and MUC5) expression into two to four subtypes; columnar and cuboidal types [19]; pancreatobiliary, intestinal, and/or gastric, and/or oncocytic types [2,6,20]. Our case was cuboidal and oncocytic types cholangiocarcinoma. Shibahara et al., concluded in their report that cuboidal type showed a better prognosis than columnar type, explaining cuboidal type was a counterpart of pancreaticobiliary type of IPMN-P and columnar type was that of intestinal type [19]. To the contrary, Zen et al., thought the oncocytic type as a variant of the pancreaticobiliary type and pointed out, instead of comment on prognosis, that only the pancreaticobiliary type was observed in non-papillary cholangiocarcinoma showing a poor prognosis compared to papillary cholangiocarcinoma [20]. Clinicopathological characteristics of oncocytic type IPMN-B have been sporadically and separately reported, with its similar features to those of papillary cholangiocarcinoma in general and its still unclearness about tumor behavior in the presence of oncocytes [11,21,22].

Over the last two decades, not a few cases on IPMN-B have been accumulated and its concept has continued to evolve: two types of intraductal biliary neoplasms, histopathological features and subtypes, resemblance to IPMN-P, diagnosis, radiologic findings and classifications, treatment strategy, surgical outcome, and prognosis. Therefore, IPMN-B deserves accepting as a discrete disease entity with a caution, being one definite type of intraductal biliary neoplasms and a biliary counterpart of IPMN-P. However, since there are still controversies on IPMN-B, more continual reports and studies are warranted to draw a full consensus on IPMN-B.

## Conclusion

We report herein a case diagnosed with typical findings of IPMN-B and successfully treated by right trisectionectomy with caudate lobectomy and extrahepatic bile duct resection. Considering a favorable prognosis of IPMN-B compared to that of non-papillary biliary neoplasms, this tumor can be a good indication for aggressive surgical resection regardless of tumor size. Furthermore, for establishing the concept of IPMN-B, more continual reports and studies are warranted.

## Consent

Written informed consent was obtained from the patient for publication of this case report and accompanying images. A copy of the written consent is available for review by the Editor-in-Chief of this journal.

## Competing interests

The authors declare that they have no competing interests.

## Authors' contributions

WJS participated in management of the patient, collecting the patient's data, and drafting the manuscript. SJ carried out management of the patient, conceived of the study, participated in its design and coordination, and helped to draft the manuscript. All authors read and approved the final manuscript.
